# The Prognostic Role of Glutathione and Its Related Antioxidant Enzymes in the Recurrence of Hepatocellular Carcinoma

**DOI:** 10.3390/nu13114071

**Published:** 2021-11-14

**Authors:** Yung-Fang Hsiao, Shao-Bin Cheng, Chia-Yu Lai, Hsiao-Tien Liu, Shih-Chien Huang, Yi-Chia Huang

**Affiliations:** 1 Department of Nutrition, Chung Shan Medical University, Taichung 40201, Taiwan; summerbreathsrelax@gmail.com (Y.-F.H.); chiayulai@vghtc.gov.tw (C.-Y.L.); chienchien2011@gmail.com (S.-C.H.); 2 Division of General Surgery, Department of Surgery, Taichung Veterans General Hospital, Taichung 40705, Taiwan; sbc@vghtc.gov.tw (S.-B.C.); langhsky1981@gmail.com (H.-T.L.); 3 School of Medicine, Chung Shan Medical University, Taichung 40201, Taiwan; 4 Department of Nutrition, Chung Shan Medical University Hospital, Taichung 40201, Taiwan; 5 Department of Health Industry Technology Management, Chung Shan Medical University Hospital, Taichung 40201, Taiwan

**Keywords:** glutathione, glutathione peroxidase, glutathione reductase, oxidative stress, hepatocellular carcinoma, recurrence

## Abstract

The imbalance of high oxidative stress and low antioxidant capacities is thought to be a significant cause of the development and progression of hepatocellular carcinoma (HCC). However, the impact of oxidative stress, glutathione (GSH), and its related antioxidant enzymes on the recurrence of HCC has not been investigated. The purpose of this study was to compare the changes to oxidative stress and GSH-related antioxidant capacities before and after tumor resection in patients with HCC recurrence and non-recurrence. We also evaluated the prognostic significance of GSH and its related enzymes in HCC recurrence. This was a cross-sectional and follow-up study. Ninety-two HCC patients who were going to receive tumor resection were recruited. We followed patients’ recurrence and survival status until the end of the study, and then assigned patients into the recurrent or the non-recurrent group. The tumor recurrence rate was 52.2% during the median follow-up period of 3.0 years. Patients had significantly lower plasma malondialdehyde level, but significantly or slightly higher levels of GSH, glutathione disulfide, trolox equivalent antioxidant capacity, glutathione peroxidase (GPx), and glutathione reductase (GR) activities after tumor resection compared to the respective levels before tumor resection in both recurrent and non-recurrent groups. GSH level in HCC tissue was significantly higher than that in adjacent normal tissue in both recurrent and non-recurrent patients. Decreased plasma GPx (HR = 0.995, *p* = 0.01) and GR (HR = 0.98, *p* = 0.04) activities before tumor resection, and the increased change of GPx (post—pre-resection) (HR = 1.004, *p* = 0.03) activity were significantly associated with the recurrence of HCC. These findings suggest there might be a possible application of GPx or GR as therapeutic targets for reducing HCC recurrence.

## 1. Introduction

Hepatocellular carcinoma (HCC) is the most common type of liver cancer, and was the sixth most commonly diagnosed cancer worldwide, with approximately 905,677 people diagnosed with liver cancer in 2020 [1]. Similar to its high incidence, the mortality of liver cancer ranked third among all types of cancer [1]. Many risk factors, such as hepatitis virus infection, cirrhosis, alcohol abuse, and cigarette exposure, might play a mediating role by stimulating chronic inflammation and increasing oxidative stress, leading to HCC development and progression [2,3]. If antioxidant capacities are insufficient to overcome the oxidative stress burden, this could promote genetic mutations in liver cells and eventually progress to liver cancer [4,5].

Glutathione (GSH) is a tripeptide, which is composed of glycine, glutamate and cysteine, and is mainly synthesized and metabolized in the liver. GSH has a thiol (-SH) group that can function as a reducing agent to supply electrons via glutathione peroxidase (GPx) to reduce organic peroxide (ROOH) to hydroxyl form of organic substance (ROH) and water, and then itself oxidizes to glutathione disulfide (GSSG). GSSG is reduced to GSH by the catalyzation of glutathione reductase (GR). GSH can also directly conjugate and detoxify electrophilic xenobiotics via glutathione *S*-transferase (GST) [6,7]. Therefore, GSH and its related enzymes (i.e., GPx, GR and GST) constitute an important endogenous antioxidant system in the human body [8,9]. Patients with HCC have been observed to have high lipid peroxidation products (i.e., malondialdehyde, MDA) and reactive oxygen metabolite levels [10,11,12], but low levels of GSH [10,13] and GPx, activities [12,14] compared to patients with hepatitis and healthy controls. On the other hand, some studies showed that the serum GSH level in HCC patients was similar to that seen in non-alcoholic fatty liver disease patients [15], and some patients even had higher plasma GSH levels than those of healthy controls [16]. Although diverse GSH levels in HCC patients have been reported among different studies, GSH and its related enzymes seem to play important roles in protecting against the development of or delaying the progression of HCC.

Liver tumor resection is currently the mainstay of curative treatment for HCC [17]. Without tumor interference, we observed that HCC patients had decreased plasma MDA and oxidized low density lipoprotein levels, and increased plasma GSH level, GPx, and GR activities when compared to levels before tumor resection [18]. The balance of oxidative stress and antioxidant capacity seems to be somewhat restored after tumor resection; however, there is a high recurrence rate of HCC after tumor resection [19,20,21]. Previous studies indicated that high oxidative stress and low antioxidant capacities in HCC tissue led to high rates of mortality and recurrence of HCC [22,23,24,25]. We speculate that oxidant and antioxidant status before tumor resection as well as their status change after tumor resection probably influence HCC prognosis. However, the association between changes in oxidative stress and antioxidant capacities and the recurrence of HCC has not yet been fully investigated. In order to obtain a clearer picture of the role of oxidative stress and the GSH-dependent antioxidant system in HCC recurrence, we aimed to compare the changes of oxidative stress and antioxidant capacities before and after tumor resection in patients with HCC recurrence and non-recurrence. Additionally, we explored the possible prognostic significance of GSH and its related enzymes in HCC recurrence.

## 2. Materials and Methods

### 2.1. Study Design and Sample Size Calculation

**Figure 1:**
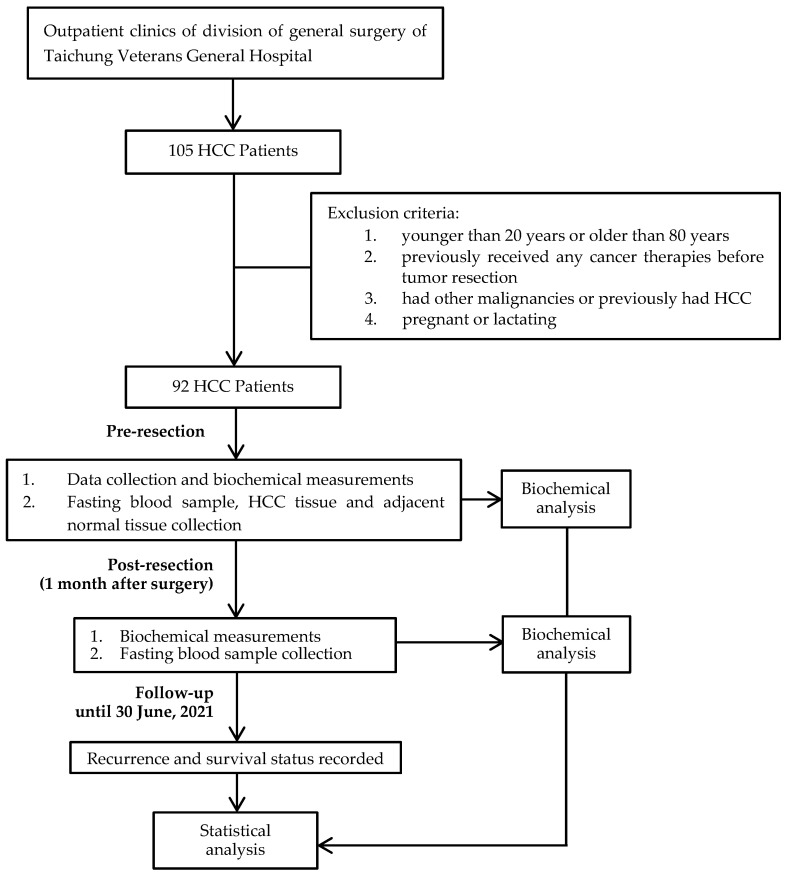
Study design and flow diagram.

This is an ongoing study, and was designed as a cross-sectional and follow-up study. Figure 1 shows the flow diagram of this study. Partial patients’ descriptions have been reported in our previous studies [18,26]. We used the Stata software (version 17.0, StataCorp LLC, College Station, TX, USA) to calculate the required sample size for the purpose of this study. According to a report by Qi et al. [27], HCC patients with plasma GPx3 levels that were less than the median value had significantly poor overall survival compared with patients whose levels were greater than the median value, with a hazard ratio of 4.5. We thus assumed that for GSH or its related antioxidant enzyme activity to be capable of significantly predicting the recurrence of HCC, a hazard ratio of 4.5 would need to be detected, with a power of 80%, a two-sided test with an α of 0.05, and a dropping rate of 20%. The calculated sample size requirement was at least 75 subjects. We finally recruited 92 HCC patients, which was greater than the required number of subjects. 

### 2.2. Patient Eligibility and Tumor Pathology

Patients were recruited from January 2014 to May 2021 if they were diagnosed with primary HCC (International Classification of Diseases 9, code 155.0) and were scheduled to undergo tumor resection. Patients were excluded if they were younger than 20 y or older than 80 y, had previously received any cancer therapies before tumor resection, had other malignancies, had previously had HCC, or were pregnant or lactating. This study was conducted in the division of general surgery of Taichung Veterans General Hospital, and was approved by the Institutional Review Board of Taichung Veterans General Hospital (IRB TCVGH No. CF17250A). Each subject provided informed consent.

An oncologist and a pathologist confirmed the patients’ diagnoses and staging based on the 7th and 8th edition of the American Joint Committee on Cancer staging system [28]. Tumor histological gradings were classified as well-, moderately-, and poorly-differentiated. Tumor number, maximum tumor size (cm), and lymph-vascular invasion status were also documented.

### 2.3. Data Collection and Biochemical Measurements

Patients’ basic characteristics were recorded on the day before tumor resection (pre-resection), and included age, gender, medication use, clinical condition (i.e., cirrhosis and hepatitis), smoking and drinking habits, and the frequency and dosage of regularly used nutritional supplements. Patients had their height, weight, and blood pressure (mmHg) measured at pre-resection and one month after tumor resection (post-resection). Height and weight were used to calculate body mass index (BMI, kg/m^2^).

Fasting blood samples were collected in vacutainer tubes (Becton Dickinson, Rutherford, NJ, USA) containing an EDTA anticoagulant and no anticoagulant, and were wrapped in tin foil to protect from UV light at pre-resection and post-resection. Serum or plasma samples were immediately separated after blood was drawn with centrifugation at 806× *g* for 10 min at 4 °C, and were preserved at −80 °C until analysis. Serum samples were used to measure alanine aminotransferase (ALT), aspartate aminotransferase (AST), α-fetoprotein, total bilirubin, albumin, high sensitivity C-reactive protein (hs-CRP), blood urea nitrogen (BUN), GSH and GSSG, while plasma samples were used to measure levels of MDA and trolox equivalent antioxidant capacity (TEAC), activities of GPx, GR and GST. 

Hepatocellular tumor tissue and adjacent normal liver tissue were obtained at the time of surgical resection. Resected tumor and adjacent normal liver tissues were homogenized in 1 mL phosphate saline buffer on ice. Homogenates were centrifuged at 8870× *g* for 10 min at 4 °C, and the supernatants were collected and stored at −80 °C until analysis. Tissue homogenates were used to measure levels of MDA, GSH, and GSSG, as well as TEAC.

Plasma and tissue MDA levels were evaluated based on thiobarbituric acid reactive substance according to the method of Lapenna et al. [29] To assess TEAC in plasma and tissue, we measured the ability of a sample to remove free radicals (ABTS^+^) to represent the TEAC level according to a method described by Arnao et al. [30] Serum and tissue GSH, GSSG levels, plasma GST activities were measured using commercial assay kits (BioVision Incorporated, Milpitas, CA, USA), and plasma GPx activities were analyzed using a different commercial brand of assay kit (Cayman Chemical Company, Ann Arbor, MI, USA). Since GR catalyzes the reduction of GSSG to GSH, using β-nicotinamide dinucleotide phosphate (NADPH) as the hydride donor, for the assessment of plasma GR activity, we measured the oxidation rate of NADPH to NADP^+^ in one minute measured at 340 nm using a spectrophotometer [31]. 

### 2.4. Follow-Up Procedure

Patients received regular image examinations (i.e., ultrasound, computed tomography (CT) or magnetic resonance imaging (MRI)) and a serum α-fetoprotein test every 3 months after tumor resection. HCC recurrence was confirmed when CT and MRI results showed intrahepatic recurrence. We followed patients from the day of tumor resection to the event (recurrence or death) or to the end of this study on 30 June 2021. The recurrence time was calculated from the day of tumor resection to the day of confirmed HCC recurrence. We assigned patients into recurrent or non-recurrent groups based on whether they had HCC recurrence at the end of this study.

### 2.5. Statistical Analysis

The SigmaPlot software (version 12.5, Systat Software Inc., Chicago, IL, USA) was used for all data analyses. Continuous variables were expressed as mean ± standard error (SE) with median in parentheses. The change value before and after tumor resection was calculated as the level of post-resection minus the level of pre-resection (level of post-resection—pre-resection, ∆). Parameter differences between pre-resection and post-resection, or between HCC tissue and adjacent normal tissue within groups utilized paired *t*-test or Wilcoxon signed rank test. Student’s *t*-test or Mann–Whitney rank sum test was used to compare values between recurrent and non-recurrent groups. Categorical variables, reported as counts and percentage, were compared with the Chi-square or Fisher’s exact test between the recurrent and the non-recurrent groups. Spearman’s correlation coefficient (*r*_s_) was used to evaluate the relationship between the level of GSH in serum and in HCC tissue. A Cox proportional hazard regression model was conducted to explore the hazard ratios (HR) of HCC recurrence according to MDA, TEAC, GSH, GSSG, GSSG/GSH ratio, GPx, GR, and GST activities with 95% confidence intervals after adjusting for potential confounders including demographic characteristics (i.e., age, gender (men, 1; women, 0), smoking and drinking habits (yes, 1; no, 0), and supplement intake (yes, 1; no, 0)), clinical condition (i.e., cirrhosis (yes, 1; no, 0)), and tumor characteristics (i.e., lymph-vascular invasion status (yes, 1; no, 0)). A two-sided *p* < 0.05 was defined as statistically significant.

## 3. Results

**Figure 2:**
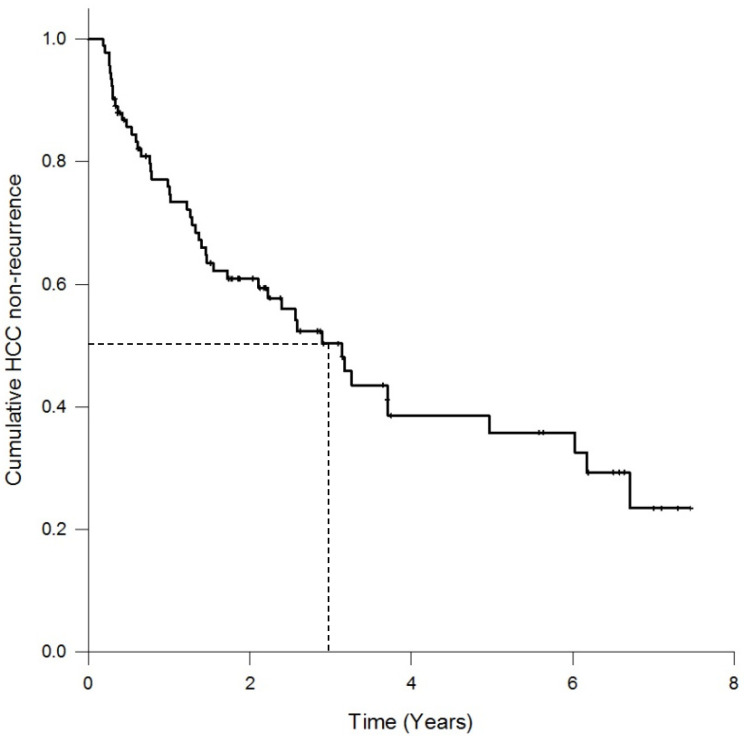
Cumulative HCC non-recurrence curve of patients with hepatocellular carcinoma (*n* = 92).

A total of 92 patients with HCC who underwent tumor resection, consisting of 75 men (81.5%) and 17 women (18.5%), participated in this study. The median follow-up period was 3 years (range, 0.3–7.5 years). The cumulative HCC non-recurrence curve is shown in Figure 2. The tumor recurrence rate was 52%, with 48 patients in the recurrent group and 44 patients in the non-recurrent group. Ten patients died after tumor recurrence.

The demographic and clinical characteristics of the patients are shown in Table 1. Fifteen percent of patients had regular alcohol consumption, 29% of patients were smokers, and 16% of patients regularly took nutritional supplements. Liver cirrhosis was observed in 25 out of 92 HCC patients (27.2%), and more than 90% of patients suffered from hepatitis B or C virus infection. The result of tumor pathology showed that 52.2% patients were in TNM stage I, and the remaining patients were in TNM stage II. Most tumor histological grades were moderately differentiated or poorly differentiated. Only 10% of patients had multifocal tumors, and 55 patients (59.8%) had lymph-vascular invasion. BMI was significantly decreased after tumor resection compared to pre-resection BMI in both recurrent and non-recurrent groups. The level of systolic blood pressure in patients with recurrence at post-resection was significantly lower than at the pre-resection level. 

Table 2 shows that serum levels of AST and α-fetoprotein were significantly reduced after tumor resection in both recurrent and non-recurrent patients. Recurrent patients had significantly increased serum total bilirubin and BUN levels, while non-recurrent patients had significantly increased serum albumin and hs-CRP levels at post-resection when compared to that at pre-resection.

Plasma/serum values of oxidative stress and antioxidant capacity indicators are listed in Table 3. Patients had significantly lower MDA levels but higher levels of GSH, GSSG, and TEAC, as well as GR activity after tumor resection, compared to that before tumor resection in both recurrent and non-recurrent groups. Post-resection GPx activity was significantly increased, while post-resection GST activity was significantly decreased compared to pre-resection activity in recurrent patients. We also analyzed indicators of oxidative stress and antioxidant capacities in HCC tissue and adjacent normal tissue (Table 4). Levels of GSH, GSSG, and TEAC in HCC tissue were significantly higher than that in adjacent normal tissue. Compared to recurrent patients, non-recurrent patients had significantly lower GSH levels in HCC tissue and adjacent normal tissue. There was a significant association between pre-section serum GSH level and GSH level in HCC tissue among all HCC patients (*r*_s_ = −0.46, *p* < 0.01), in recurrent patients (*r*_s_ = −0.47, *p* < 0.01), and in non-recurrent patients (*r*_s_ = −0.44, *p* < 0.01). 

Since oxidative stress and antioxidant capacities might affect HCC recurrence, we calculated the HR of HCC recurrence according to MDA, GSH, GSSG, GSSG/GSH ratio, and GSH-related antioxidant enzyme activities after adjusting for age, gender, smoking and drinking habits, cirrhosis, supplement intake, and lymph-vascular invasion status. We observed that pre-resection plasma GPx activity (HR = 0.995, 95% CI = 0.991–0.999, *p* = 0.01) and plasma GR activity (HR = 0.98, 95% CI = 0.956–0.999, *p* = 0.04) could predict HCC recurrence. In addition, both the change of plasma GPx activity (post—pre-resection level) (HR = 1.004, 95% CI = 1.00–1.01, *p* = 0.03) and the change of plasma TEAC level (post—pre-resection level) (HR = 1.001, 95% CI = 1.000–1.001, *p* = 0.02) were significantly associated with recurrence of HCC. However, the post-resection values of plasma MDA, GSH, and its related antioxidant enzyme activities, as well as tissue levels of MDA, GSH, GSSG, and TEAC were not significantly associated with HCC recurrence.

## 4. Discussion

Previous studies have shown that HCC patients had higher plasma levels of oxidative stress markers, and lower plasma levels of GSH and GSH-related antioxidant enzymes when compared to healthy controls, hepatitis patients, or liver cirrhotic patients [11,12,15]. Since the imbalance of high oxidative stress and low antioxidant capacities is a significant cause of the development and progression of HCC, it is not too surprising that our HCC patients exhibited a higher oxidative stress status and lower antioxidant capacities before receiving tumor resection. In addition to MDA being an oxidative stress marker in the present study, the ratio of GSSG/GSH was also shown to be an oxidative stress indicator in this study. Under stable physiological conditions, the liver preserves a high concentration of GSH, and the ratio of GSSG and GSH are maintained in a narrow range of 1:10–100. When facing extensive oxidative stress or insufficient capacity of GR, the GSSG/GSH ratio would decrease to 1:1, indicating that the GSSG to GSH ratio could be used as a redox status marker [9,32]. In vitro and in vivo studies have demonstrated that the GSSG/GSH ratio can serve as a reliable oxidative stress indicator in hepatic injury research [33,34,35]. Although our results did not show a significant difference in the change of GSSG/GSH ratio before and after tumor resection in both recurrent and non-recurrent patients, we observed a higher GSSG/GSH ratio at the pre-resection in recurrent patients compared to ratios in non-recurrent patients, and this difference approached statistical significance. Studies conducted by Lorente et al. [36,37] revealed that HCC patients who underwent liver transplantation and survived for one year had a lower MDA, but had higher TEAC levels at pre-transplantation than levels in non-surviving patients. Regarding the performance of antioxidant capacity in recurrent and non-recurrent patients, our recurrent patients had lower plasma GPx and GR activities, and higher GST activities compared to levels in non-recurrent patients. Consistent with our findings, previous studies showed that patients with recurrent HCC after tumor resection [38] or after liver transplantation [27] had lower plasma GPx3 levels than those in patients without HCC recurrence. Based on our results and other findings reported in the literature, HCC patients with a relatively higher oxidative burden before tumor resection might be prone to HCC recurrence after tumor resection. Among GSH-related antioxidant enzymes in plasma, the change in performance of GST activity during pre- and post-resection was the opposite of the changes of GR and GPx activities found in this study. This phenomenon implies that GPx, which functions as a phase I antioxidant enzyme, has been exhausted owing to the presence of HCC tumor, whereas GST, which is a phase II antioxidant enzyme, took over the protective role of phase I enzyme to cope with the increased oxidative stress. Thereafter, GPx and GST activity in plasma might shift back to their original antioxidant role after tumor interference has disappeared. 

In terms of oxidative stress and antioxidant capacities expressed in HCC tissue and adjacent normal tissue, previous studies (including ours) observed lower or similar oxidative stress status and high antioxidant capacities in HCC tissue compared to adjacent normal tissue [18,23,39,40]. Furthermore, liver GSH levels in recurrent patients were higher than levels in non-recurrent patients. GSH plays an important role in HCC tumor differentiation, proliferation, progression, and metastasis [8,9,41]. Since we observed a negative association between GSH in serum and in HCC tissue, there might be a redistribution of GSH from serum to HCC tissue. Moreover, up-regulation of GSH-related transport and synthesis enzymes (i.e., γ-glutamyl transferase, glutamate cysteine ligase, and GSH synthetase) were detected in HCC cells [8,42,43]. Hence, HCC tissue could efficiently uptake GSH from the circulation to enhance its own growth, protecting itself from high oxidative damage or inducing resistance to chemotherapy [8,41,43]. However, some studies found HCC tissue had higher oxidative stress but lower antioxidant capacities compared to adjacent normal tissue [13,38,44]. The question as to the status of GSH and its related antioxidant enzymes in tumor and normal tissues requires further investigation. 

Tumor resection is one of the curative treatments for HCC. However, the recurrence rate remains high. There was an approximate recurrence rate of 50% in 3 years after tumor resection [19,20,21]. The 3-year recurrence rate in our HCC patients was 52% after tumor resection, and the 5-year recurrence rate was previously reported to be almost 70% [45]. It is still a considerable challenge to reduce the high HCC recurrence rate in clinical settings. Different prognostic markers, such as a high plasma level of α-fetoprotein before tumor resection, poor tumor characteristics, advanced tumor stage, and lymph-vascular tumor invasion have been documented to predict HCC recurrence and mortality [21,46,47]. However, attempts to reduce HCC recurrence by altering these prognostic parameters in advance have been unsuccessful. Plasma GPx and GR activities not only significantly changed before and after tumor resection, but their low activities at pre-resection were also valuable markers for predicting poor HCC prognosis. GPx oxidizes GSH to GSSG, while GR reduces GSSG to GSH; the synergic action of these two enzymes could predict the recurrence of HCC. Similar to our findings, Qi et al. [38] reported that patients with a lower plasma GPx3 level had an inferior 5-year recurrence rate compared to patients with a higher plasma GPx3 level. Decreased systemic GPx3 levels have been shown to promote colon tumor initiation and carcinogenic stimuli in animal studies [48]. In contrast to the association of plasma GPx with clinical outcomes, high GPx2 expression in patients’ HCC tissue was positively rather than negatively correlated with α-fetoprotein level, tumor size, and HCC recurrence [39]. The activities of GPx in plasma and tissue are probably interrelated and result in opposing actions, leading to different clinical outcomes. Unfortunately, we did not measure the GPx activity in HCC tissue, and thus we were unable to determine the association between tissue GPx level and HCC recurrence in this study. In addition to investigating individual values at pre-resection, we also found that the increase in the level of GPx between pre- and post-resection significantly increased the HCC recurrence risk. Taken together, the findings of previous investigations and the results of the present study suggest that HCC patients with greater depletion of GPx at pre-resection to counterbalance the tumor burden and with greater repletion of GPx after tumor resection had a higher likelihood of HCC recurrence. Regardless of the performance of GPx activity in HCC tissue, plasma GPx seemed to be involved in HCC development and could be used as a prognostic factor for HCC recurrence. Unfortunately, we could not further find an appropriate cut-off value for plasma GPx and GR activities to better predict the HCC recurrence. Further studies are warranted to seek a diagnostic value of plasma GPx or GR activities for HCC recurrence, and to understand the mechanistic role that GPx plays in HCC recurrence.

The strength of this study was the combination of its cross-sectional and follow-up design, which enabled an exploration of the changes in oxidative stress and antioxidant capacities before and after tumor resection in relation to HCC recurrence. In addition, the results may help to gain a better understanding of oxidative stress and antioxidant capacities since plasma and tissue samples were simultaneously obtained in the present study. There were some limitations to our study. We did not measure erythrocyte levels of GSH and GSSG in this study. Otherwise, the possibility of redistribution of plasma and erythrocyte levels could be evaluated. Limited HCC tissue and adjacent normal tissues were available and thus we were only able to measure GSH and GSSG, and it was not possible to assess GSH-related antioxidant enzyme activities. Another limitation was that we only measured MDA levels and GSSG/GSH ratios as oxidative stress markers, although other measures of oxidative stress could be considered in future research to confirm the correlation between oxidative stress and HCC recurrence. In addition, the patient population was limited to HCC stage I and II patients who were scheduled to undergo tumor resection, and thus our observations may not be applicable to patients with advanced cancer stage or receiving other therapy treatments (e.g., radiofrequency ablation and transcatheter arterial chemoembolization).

## 5. Conclusions

Both HCC recurrent and non-recurrent patients had similar changes of oxidative stress and antioxidant capacities, when comparing before and after tumor resection. However, HCC patients who had a lower plasma GPx and GR activity before tumor resection had a higher HCC recurrence rate. These results imply that GPx or GR may have potential as therapeutic targets for reducing HCC recurrence.

## Figures and Tables

**Table 1:** Characteristics of patients with recurrent and non-recurrent hepatocellular carcinoma at pre-resection and post-resection ^1^.

Characteristics	Recurrence (*n* = 48)		Non-Recurrence (*n* = 44)	
	Pre-Resection	Post-Resection	Pre-Resection	Post-Resection
Age (years)	60.9 ± 9.7		58.6 ± 10.3	
Gender (male/female)	39/9		36/8	
BMI (kg/m^2^)	24.0 ± 3.0	23.6 ± 2.8 ^2,^*	24.9 ± 2.8	24.5 ± 2.4 ^3,^*
Blood pressure				
SBP (mmHg)	131.3 ± 16.2	126.7 ± 18.6 ^2,^*	126.8 ± 13.4	129.8 ± 16.8 ^3^
DBP (mmHg)	76.4 ± 11.2	73.6 ± 13.3 ^2^	77.5 ± 11.6	79.2 ± 11.4 ^3^
Smoking habit (*n*, %)				
Yes	13 (27.1%)		14 (31.8%)	
No	35 (72.9%)		30 (68.2%)	
Drinking habit (*n*, %)				
Yes	6 (12.5%)		8 (18.2%)	
No	42 (87.5%)		36 (81.8%)	
Cirrhosis (*n*, %)				
Yes	17 (35.4%)		7 (15.9%)	
No	31 (64.6%)		37 (84.1%)	
Hepatitis (*n*, %)				
No hepatitis	3 (6.3%)		3 (6.8%)	
Hepatitis B	28 (58.3%)		30 (68.2%)	
Hepatitis C	16 (33.3%)		10 (22.7%)	
Co-hepatitis B and C	1 (2.1%)		1 (2.3%)	
Use of nutritional supplement				
Yes	10 (20.8%)		5 (11.4%)	
No	38 (79.2%)		39 (88.6%)	
Cancer stage (*n*, %)				
Stage I	27 (56.3%)		21 (47.7%)	
Stage II	21 (43.8%)		23 (52.3%)	
Histological grading (*n*, %)				
Well-differentiated	1 (2.1%)		0 (0%)	
Moderately differentiated	21 (43.8%)		25 (56.8%)	
Poorly differentiated	26 (54.2%)		19 (43.2%)	
Tumor number (*n*, %)				
Solitary	39 (81.3%)		43 (97.7%)	
Multifocal	9 (18.8%)		1 (2.3%)	
Tumor size (*n*, %)				
≤5 cm	34 (70.8%)		27 (61.4%)	
>5 cm	14 (29.2%)		17 (38.6%)	
Lymph-Vascular Invasion (*n*, %)				
Absent	33 (68.8%)		22 (50%)	
Present	15 (31.3%)		22 (50%)	

^1^ Values are means ± standard error. BMI, body mass index; SBP, systolic blood pressure; DBP, diastolic blood pressure. ^2^ *n* = 24. ^3^ *n* = 33. * Values are significantly different between pre-resection and post-resection within group (*p* < 0.05).

**Table 2:** Hematological measurements in patients with recurrent and non-recurrent hepatocellular carcinoma at pre-resection and post-resection ^1^.

Parameters	Recurrence (*n* = 48)				Non-Recurrence (*n* = 44)			
	Pre-Resection	Post-Resection	*p* Value ^2^	Δ(Post—Pre-Resection)	Pre-Resection	Post-Resection	*p* Value ^2^	Δ(Post—Pre-Resection)
ALT (U/L)	64.4 ± 8.2 (43.5)	54.2 ± 7.2 (39.5)	0.12	−10.3 ± 8.0 (−3.0)	76.4 ± 15.6 (37.0)	44.2 ± 4.7 (35.5)	0.10	−33.9 ± 16.5 (−2.0)
AST (U/L)	63.0 ± 8.7 (36.5)	42.8 ± 5.6 (31.5)	0.01	−20.2 ± 9.0 (−5.5)	77.5 ± 15.4 (37.0)	37.3 ± 3.5 (30.0)	0.003	−42.9 ± 16.4 (−5.5)
α-fetoprotein (ng/mL)	1240.1 ± 941.8 (19.1)	34.1 ± 10.0 (9.8)	<0.001	−1205.3 ± 937.4 (−3.1)	2562.8 ± 1504.8 (31.3)	119.8 ± 67.0 ^#^ (5.7)	<0.001	−2652.5 ± 1594.0 (−21.8)
Total bilirubin (mg/dL)	0.6 ± 0.04 (0.6)	1.1 ± 0.3 ^3^ (0.8)	0.01	0.5 ± 0.3 (0.2)	0.7 ± 0.1 (0.6)	0.7 ± 0.1 ^4^ (0.6)	0.58	0.02 ±0.03 (0.0)
Albumin (g/dL)	4.1 ± 0.1 (4.1)	4.1 ± 0.1 ^3^ (4.1)	0.69	−0.01 ± 0.8 (0.1)	4.1 ± 0.1 (4.1)	4.3 ± 0.04 ^#^ (4.3)	0.004	0.2 ± 0.1 ^†^ (0.1)
hs-CRP (mg/dL)	0.9 ± 0.3 (0.2)	0.7 ± 0.1 (0.4)	0.07	−0.2 ± 0.3 (0.1)	0.4 ± 0.1 (0.1)	0.5 ± 0.2 (0.3)	0.01	0.1 ± 0.2 (0.1)
BUN (mg/dL)	14.3 ± 0.8 (13.0)	16.1 ± 0.9 (15.0)	0.01	1.7 ± 0.7 (2.0)	14.6 ± 0.9 (13.5)	16.1 ± 0.9 (15.0)	0.49	0.6 ± 0.8 (0.5)

^1^ Values are means ± standard error with median in parentheses. ALT, alanine aminotransferase; AST, aspartate aminotransferase; hs-CRP, high sensitivity C-reactive protein; BUN, blood urea nitrogen. ^2^ The comparison of value at pre-resection and at post-resection within group. ^3^ *n* = 24. ^4^ *n* = 33. ^#^ Values are significantly different from recurrence group at post-resection (*p* < 0.05). ^†^ Values are significantly different to the recurrence group in ∆(Post—Pre-resection) (*p* < 0.05).

**Table 3:** Indicators of plasma/serum oxidative stress and antioxidant capacities in patients with recurrent and non-recurrent hepatocellular carcinoma at pre-resection and post-resection ^1^.

Parameters	Recurrence (*n* = 48)				Non-Recurrence (*n* = 44)			
	Pre-Resection	Post-Resection	*p* Value ^2^	Δ(Post—Pre-Resection)	Pre-Resection	Post-Resection	*p* Value ^2^	Δ(Post—Pre-Resection)
Oxidative stress marker								
MDA (μmol/L)	0.9 ± 0.04 (0.9)	0.8 ± 0.5 (0.7)	0.01	−0.1 ± 0.1 (−0.2)	1.0 ± 0.04 (1.0)	1.0 ± 0.2 (0.8)	0.01	0.0 ± 0.1 (−0.1)
GSSG/GSH ratio	34.6 ± 20.1 (12.3)	24.8 ± 7.6 (8.8)	0.20	−12.5 ± 17.4 (−1.9)	17.6 ± 4.1 (9.4)	23.5 ± 13.0 (7.7)	0.10	6.7 ± 12.5 (−1.2)
Antioxidant capacities								
GSH (μmol/L)	54.0 ± 5.2 (47.6)	67.9 ± 6.8 (62.9)	0.01	14.9 ± 5.5 (12.2)	64.8 ± 5.9 (60.9)	83.8 ± 8.3 (77.9)	0.003	17.6 ± 6.1 (13.2)
GSSG (μmol/L)	555.1 ± 15.4 (575.7)	610.1 ± 16.5 (606.0)	0.01	52.5 ± 17.6 (50.6)	552.7 ± 15.6 (554.1)	611.1 ± 18.4 (593.9)	<0.001	58.1 ± 12.7 (37.7)
GPx (nmol min^−1^ mL^−1^)	130.9 ± 13.4 (132.4)	152.3 ± 11.2 (160.5)	0.03	22.4 ± 14.5 (15.3)	144.5 ± 7.4 (149.0)	157.4 ± 9.4 (175.7)	0.17	16.5 ± 10.2 (15.3)
GR (nmol min^−1^ mL^−1^)	54.8 ± 2.4 (54.7)	69.2 ± 2.7 (67.4)	<0.001	14.1 ± 2.7 (9.6)	64.7 ± 3.2 * (59.6)	70.4 ± 2.9 (70.8)	0.047	5.7 ± 3.7 (7.8)
GST (nmol min^−1^ mL^−1^)	38.7 ± 4.1 (35.8)	30.0 ± 3.0 (25.8)	0.03	−8.9 ± 4.3 (−6.0)	24.7 ± 3.1 * (15.2)	22.1 ± 2.8 (18.2)	0.31	−2.6 ± 2.7 (−3.0)
TEAC (μmol/L)	4346.4 ± 83.3 (4334.7)	4592.6 ± 65.7 (4602.6)	0.01	269.2 ± 101.1 (318.0)	4394. 9 ± 76.0 (4401.3)	4610.1 ± 69.5 (4642.1)	0.02	215.2 ± 92.0 (219.1)

^1^ Values are means ± standard of error with median in parentheses. MDA, malondialdehyde; GSH, glutathione; GSSG, glutathione disulfide; GPx, glutathione peroxidase; GR, glutathione reductase; GST glutathione *S*-transferase; TEAC, trolox equivalent antioxidant capacity. ^2^ The comparison of value at pre-resection and at post-resection within group. * Values are significantly different from recurrence group at pre-resection (*p* < 0.05).

**Table 4:** Indicators of oxidative stress and antioxidant capacities of liver tissue in patients with recurrent and non-recurrent hepatocellular carcinoma ^1^.

Characteristics	Recurrence (*n* = 48)		Non-Recurrence (*n* = 44)	
	Adjacent Normal Tissue	HCC Tissue	Adjacent Normal Tissue	HCC Tissue
Oxidative stress marker				
MDA (μmol/g protein)	0.7 ± 0.1 (0.7)	0.5 ± 0.1 (0.3)	0.6 ± 0.1 (0.4)	0.7 ± 0.1 (0.5)
GSSG/GSH ratio	14.5 ± 1.8 ^2^ (14.1)	16.4 ± 3.4 ^2^ (13.8)	21.9 ± 2.8 ^3,#^ (19.8)	23.9 ± 3.6 ^3^ (18.2)
Antioxidant capacities				
GSH (μmol/g protein)	20.4 ± 2.4 (21.4)	30.6 ± 3.9 * (26.5)	14.5 ± 3.2 ^#^ (3.3)	20.0 ± 3.9 *^,#^ (6.6)
GSSG (μmol/g protein)	33.5 ± 1.5 ^2^ (32.5)	47.0 ± 4.4 ^2,^* (43.1)	38.7 ± 2.1 ^3^ (35.7)	54.0 ± 4.7 ^3,^* (48.4)
TEAC (μmol/g protein)	214.1 ± 9.7 (209.6)	268.3 ± 16.5 * (262.7)	202.6 ± 11.6 (189.3)	247.8 ± 17.0 * (218.7)

^1^ Values are means ± standard error with median in parentheses. MDA, malondialdehyde; GSH, glutathione; GSSG, glutathione disulfide; TEAC, trolox equivalent antioxidant capacity. ^2^ *n* = 13. ^3^ *n* = 23. * Values are significantly different from adjacent normal tissue (*p* < 0.05). ^#^ Values are significantly different from recurrence group (*p* < 0.05).

## Abbreviations

The following abbreviations are used in this manuscript:

ALT	alanine aminotransferase
AST	aspartate aminotransferase
BMI	body mass index
BUN	blood urea nitrogen
GSH	glutathione
GPx	glutathione peroxidase
GR	glutathione reductase
GST	glutathione *S*-transferase
GSSG	glutathione disulfide
HCC	hepatocellular carcinoma
hs-CRP	high-sensitivity C-reactive protein
MDA	malondialdehyde
TEAC	trolox equivalent antioxidant capacity
